# 伴TRIM24/FGFR1和FGFR1/TRIM24融合基因阳性8p11骨髓增殖综合征1例

**DOI:** 10.3760/cma.j.issn.0253-2727.2022.08.017

**Published:** 2022-08

**Authors:** 吾甫尔 古再丽努尔, 一淳 王, 利 安, 敏 毛

**Affiliations:** 新疆维吾尔自治区人民医院血液科，乌鲁木齐 830000 Department of Hematology, People's Hospital of Xinjiang Uygur Autonomous Region, Urumqi 830000, China

患者，男，71岁，2021年6月因“刺激性咳嗽1个月”住院治疗。因入院血常规显示“白细胞增高、贫血、血小板减少”而转入血液科。查体：贫血貌，全身未见皮疹、瘀点，浅表淋巴结不大，双肺呼吸音稍粗，心律齐、无杂音，腹软，肝脾肋缘下未触及。血常规：WBC 22.63×10^9^/L，HGB 97 g/L，PLT 28×10^9^/L，中性粒细胞11.61×10^9^/L，淋巴细胞1.58×10^9^/L，单核细胞6×10^9^/L，嗜酸性粒细胞1.59×10^9^/L；肝肾功能、血电解质、心肌酶正常，肿瘤标志物正常，ANA、dsDNA、ENA、ANCA阴性，血清IgE阴性。腹部B超：肝脾不大。胸部CT：两下肺散在微小结节。骨髓象：有核细胞增生明显活跃，粒系占72.5％，各阶段细胞均可见，早幼粒细胞比值增高，部分粒细胞可见核浆发育不平衡、内外浆、空泡，可见少数巨幼变、巨多分叶及假P-H畸形细胞，嗜酸性粒细胞占30％，易见幼稚嗜酸性粒细胞，嗜碱性粒细胞可见；红系占16.5％，以中晚幼红细胞为主，可见核固缩、胞浆量少，偶见花瓣核、嗜碱性点彩幼红细胞，成熟红细胞大小不等；全片巨核细胞14个，其中裸巨核3个，颗粒巨核11个，巨核细胞形态未见明显异常，淋巴细胞占1.5％；单核细胞占9％。血片：嗜酸性粒细胞可见，嗜碱性粒细胞占比增高，单核细胞占比明显增高，形态未见明显异常。分类100个白细胞可见有核红细胞4个。诊断意见：考虑为慢性粒-单核细胞白血病伴嗜酸性粒细胞增多。骨髓流式细胞术检测：单核细胞占有核细胞的18％（以成熟单核细胞为主），中性粒细胞占有核细胞的53.5％（部分细胞存在发育异常），嗜酸性粒细胞占有核细胞的13％，淋巴细胞占有核细胞的6％（亚群分布大致正常）；骨髓细胞染色体核型：46,XY,N[15]；骨髓病理：骨髓造血组织增生不均一，增生区粒系中晚幼以上阶段细胞比例增高，红系增生减低，巨核细胞偏少，单核细胞散在易见；FISH检测FGFR1分离探针可见信号分离，阳性率为87％。髓系血液病34种高频基因突变筛查：U2AF1NM_006758.2chr21:44524456c.101C>Tp.Ser34Phe：49.6％。BCR-ABL（p190、210、230）均阴性。JAK2V617F、MPL、CALR基因阴性。TCR、IgH基因阴性。FIP1L1/PDGFRα、ETV6-PDGFRβ、PCM1/JAK2阴性。全外显子基因测序检出TRIM24/FGFR1融合基因阳性（该融合由TRIM24基因9号外显子和FGFR1基因10号外显子重组形成），TRIM24/FGFR1和FGFR1/TRIM24融合基因阳性。U2AF1基因上检测到错义突变。结合患者临床表现和实验室检查结果诊断为“伴TRIM24/FGFR1和FGFR1/TRIM24融合基因阳性8p11骨髓增殖综合征”，于2021年6月开始予以阿扎胞苷联合维奈克拉诱导化疗，化疗第21天复查骨髓象：粒系占27.5％，以成熟粒细胞为主，红系占9.5％，巨核细胞少见，原始细胞占2.5％。骨髓流式细胞术：原始细胞占有核细胞的0.1％，单核细胞占有核细胞的1.5％（表型成熟），粒细胞占有核细胞的27.9％（未见明显发育异常）。骨髓涂片和流式细胞术检查提示获得完全缓解（CR）。随访至2021年8月病情稳定，未复发。

讨论：本例患者骨髓涂片和骨髓病理均显示嗜酸性粒细胞比值增高、幼稚嗜酸性粒细胞易见、单核细胞比值增高，染色体核型46,XY,N[15]，CMML相关TET2、SRSF2、ASXL1、SETBP1等基因阴性，PDGFRA、PDGFRB、PCM1-JAK2阴性。FISH检测示FGFR1基因重排。患者并没有典型的8号染色体，血液肿瘤全转录组测序U2AF1基因检测到错义突变、TRIM24/FGFR1和FGFR1/TRIM24融合基因阳性，国内尚未见报道。

